# The gut microbiota and its metabolites: novel therapeutic targets for inflammatory bowel disease

**DOI:** 10.3389/fimmu.2025.1690279

**Published:** 2025-11-24

**Authors:** Xingyu Shen, Yue Li, Deqiang Wang, Kang Sun

**Affiliations:** 1 Department of Gastrointestinal Surgery, Affiliated Hospital of Jiangsu University, Zhenjiang, Jiangsu, China; 2 Medical School of Jiangsu University, Jiangsu University, Zhenjiang, Jiangsu, China; 3 Department of Oncology, Affiliated Hospital of Jiangsu University, Zhenjiang, Jiangsu, China

**Keywords:** inflammatory bowel disease, gut microbiota, short-chain fatty acids, bile acids, tryptophan

## Abstract

Inflammatory bowel disease (IBD) pathogenesis is critically influenced by gut microbiota dysbiosis and perturbations in associated metabolites. This review outlines current IBD diagnostic and therapeutic paradigms, highlighting the persistent focus on the management of inflammatory symptoms and the absence of curative interventions. We elucidate the mechanistic links between gut microbiota dysregulation and IBD progression, with an emphasis on the immunomodulatory functions of microbial metabolites—specifically short-chain fatty acids (SCFAs), bile acids (BAs), and tryptophan (Trp) metabolism—in maintaining intestinal barrier integrity and attenuating inflammation. Furthermore, we evaluate microbiota-targeted therapeutic strategies, including probiotics, fecal microbiota transplantation (FMT), and metabolite-based interventions, as novel approaches for IBD management. This synthesis aims to inform future therapeutic development and accelerate the clinical translation of microbiota-modulating regimens.

## 1 Introduction

The gastrointestinal tract of humans is the habitat of a wide range of microbial species, including bacteria, fungi, viruses, and archaea, which form a complex ecosystem that is capable of coevolving with the host over time (1). Their number is extremely large, approximately 40 trillion (2). The human host and gut microbiota maintain a symbiotic relationship. In one aspect, the human body provides suitable environmental conditions for the gut microbiota to selectively colonize. In another aspect, the gut microbiota and its metabolites help the human host maintain health by strengthening the intestinal barrier and regulating immune system development, in addition to supporting other physiological processes. The abundance and composition of the gut microbiota are strongly associated with human well-being and disorders and significantly contribute to the origin and progression of multiple conditions, including IBD. IBD describes a group of chronic, nonspecific inflammatory conditions that impact the gastrointestinal tract and includes mainly ulcerative colitis (UC) and Crohn's disease (CD). Both of these conditions may manifest as abdominal distension, abdominal pain, mucopurulent or bloody stools, etc., but the anatomical site of their occurrence differs; UC is usually limited to the mucosal layer of the colon, whereas CD can cause inflammation anywhere in the gastrointestinal tract (3). The incidence and prevalence of IBD in China have increased significantly over the past 30 years (4). At present, as a newly industrialized country, China is experiencing a period of rapid growth in the incidence of IBD; if this trend continues, the incidence of IBD in China is projected to reach 12–26 cases per 100,000 people within the next 30 years, imposing a significant societal and health care burden (5). The exact etiology of IBD is still unclear, and several contributing factors, such as genetic susceptibility, environmental factors, immune dysregulation, and changes in the balance of the gut microbiota, are involved in the progression of IBD (6). Among these factors, the influence of the gut microbiota is key to triggering IBD, but the precise mechanism remains unclear. In recent years, the influence of the gut microbiota on the development, screening and diagnosis, and therapeutic amelioration of IBD has attracted increasing attention and has become one of the hotspots of current research. Here, we review the involvement of the gut microbiota and its metabolites in the development and clinical progression of IBD and their translational application to the prevention, management, and eventual cure for IBD.

## 2 Review methodology

This narrative review systematically examines the role of the gut microbiota and its metabolites in IBD. Literature was retrieved from the PubMed and Web of Science databases using keywords such as “inflammatory bowel disease”, “gut microbiota”, “short-chain fatty acids”, “bile acids”, “tryptophan”, “probiotics”, “prebiotics”, “synbiotics”, “fecal microbiota transplantation”, and Boolean operators (“AND/OR”). The search covered the period from 2001 to 2025, with a particular emphasis on studies published after 2020 to incorporate the latest advances.

The inclusion criteria comprised original studies, reviews, and meta-analyses addressing the relationships between the gut microbiota, short-chain fatty acids, bile acids, or tryptophan metabolism and the pathogenesis, diagnosis, or treatment of IBD. Clinical studies involving human patients with IBD were prioritized. Animal and *in vitro* studies were selectively included to elucidate the underlying mechanisms. The exclusion criteria included conference abstracts, non-English publications, and articles not directly relevant to the topic.

In addition to the electronic database search, we manually screened the reference lists of the retrieved articles to identify additional relevant studies. The most comprehensive or recent publication was selected for overlapping cohorts to avoid redundancy. Non-IBD studies were included only when they were deemed informative on the IBD mechanism.

## 3 Current status of IBD diagnosis and treatment

IBD encompasses a range of chronic and recurring intestinal disorders that not only lead to physical pain but also significantly affect the mental well-being and social life of patients. Therefore, timely detection and an accurate diagnosis with effective therapeutic measures are crucial for improving the overall life experience of patients. Endoscopy serves a critical function in the diagnosis and monitoring of IBD, but existing endoscopic scoring systems have limitations in clinical application. These limitations include complex procedures, a time-consuming nature, variation among observers, and a lack of uniform consensus on the definition of disease severity (7). Due to its noninvasive nature, transabdominal ultrasound (TAUS) has become the first method for examination, and its ability to screen for lesions and diagnose complications is comparable to that of other imaging methods, such as CT or MRI. However, the greatest disadvantage of TAUS is its lack of objectivity, and some pressing issues still need to be resolved in terms of universal application and standardization (8). Although traditional biomarkers, such as C-reactive protein (CRP) and fecal calprotectin (FC), can be utilized to assess the degree of inflammation, they are not specific markers of IBD and still face challenges in terms of setting valid diagnostic thresholds (9). A recent study developed a noninvasive disease severity index (DSI) using fecal calprotectin (DSI-fCal) and fecal myeloperoxidase (DSI-fMPO) as alternatives to colonoscopy. However, due to the small and restricted sample size, further large-scale studies of diverse populations is needed to validate its effectiveness and reliability (10).

Traditional treatment for IBD relies on medications for symptom control, including aminosalicylates and corticosteroids, and surgery, if necessary. Although medication is highly effective, its use is linked to several side effects. For example, sulfasalazine has long been known to cause hemolytic anemia (11). Corticosteroids cause diabetes (12). Monoclonal antibodies increase the risk of opportunistic infections (13). In addition, the diversity and complexity of IBD results in significant interindividual variation in the treatment response. Approximately 40% of patients do not respond to the first treatment, while between 13% and 46% of the remaining patients still experience a gradual loss of treatment efficacy over the next year, with valuations that may vary depending on the treatment and disease subtype (14). Although many options are emerging for the treatment of IBD, treatment outcomes have not yet reached optimal levels, and a ‘therapeutic ceiling’ has been observed in the treatment process (15); this finding highlights the need for new therapeutic targets.

As a result of progress in high-throughput sequencing technology, researchers have observed substantial shifts in the gut microbiota of IBD patients. The gut microbiota has been recognized as a potential biomarker for IBD (9). These findings have triggered a keen interest in the promising applications of noninvasive microbial markers in determining the disease diagnosis and prognosis.

## 4 The gut microbiota and IBD

### 4.1 Gut microbiota functions

In the human intestinal tract, the gut microbiota is mostly composed of Firmicutes, Bacteroidetes, Proteobacteria, and Actinobacteria, of which Firmicutes and Bacteroidetes dominate the gut microbiota and are crucial for sustaining intestinal health in humans (16), and intestinal health plays a vital role in overall human well-being. The gut microbiota can maintain intestinal health and function through a variety of physiological functions.

The gut microbiota can participate in the metabolism and absorption of many nutrients, such as sugars, fats, and proteins. For example, the intestinal tract cannot directly absorb complex dietary polysaccharides, whereas the gut microbiota can encode a substantial quantity of carbohydrate-active enzymes that reassemble and breakdown polysaccharides, assisting the human digestive system in degrading carbohydrates and breaking down indigestible dietary fiber into SCFAs, which serve key functions in supporting immune health and general wellness, among other processes (17). Some components of the gut microbiota can also metabolize proteins into amino acids, contributing to the transformation of amino acids in the gut into biogenic amines and immunoregulatory compounds (17). The ileum, the primary site for bile salt absorption, harbors a gut microbiota that participates in lipid emulsification and bile salt metabolism through enzymes such as bile salt hydrolases (BSH) and hydroxysteroid dehydrogenases; these enzymes facilitate the enterohepatic circulation and metabolism of bile salts (18).

The gut microbiota can affect an organism’s immune response, thereby inhibiting the attack of pathogens and enhancing host immunity. Immune system maturation relies on the existence of the microbiota, a requirement that was first documented in animals raised in a sterile environment. Germ-free animals exhibit a variety of intestinal immunodeficiencies, including dysplasia of gut-associated lymphoid tissue (GALT), reduced amounts of secretory immunoglobulin (sIg), and reduced numbers of CD8^+^ T cells within the epithelium (19). Components of the gut microbiota, such as *Citrobacter rodentium*, stimulate the production of intestinal T helper 17 (Th17) cells by adhering to the intestinal epithelium and that the adherence of the microbiota to the intestinal epithelium correlates with an increase in the number of intestinal IgA^+^ cells (20).

The gut microbiota is also involved in the formation of the intestinal microbial barrier, which physically restricts the proliferation of foreign pathogenic bacteria and toxins by tightly integrating with the intestinal mucosa, controlling their levels in the intestinal lumen, and inhibiting their colonization and proliferation (21). Various microorganisms contribute to preserving the structural integrity of the intestinal epithelial barrier by enhancing intercellular junctions and aiding in epithelial repair (22). The various components of the gut microbiota interact with each other to achieve dynamic equilibrium, thus maintaining homeostasis in the human body.

In summary, the gut microbiota plays a pivotal role in maintaining intestinal health and overall well-being through its involvement in nutrient metabolism, immune system modulation, and the preservation of the intestinal barrier. The metabolites produced by the gut microbiota also significantly affect disease.

### 4.2 Gut microbiota dysbiosis and IBD

We usually refer to variations in the composition and structure of the gut microbiota as intestinal dysbiosis. When IBD occurs, patients usually exhibit a gut microbiota imbalance with notable decreases in abundance, homogeneity, and biodiversity (**Table 1**). Additionally, the bacteria, viruses, and fungi in the gut microbiota differ from those found in healthy individuals. In a study of six IBD patients and six healthy controls, Manichanh et al. (23) observed a notable decrease in colonic microbial diversity among IBD patients compared with healthy individuals, as determined through 16S rRNA sequencing of fecal samples from all 12 participants. Another study similarly demonstrated that patients with IBD show marked quantitative and qualitative changes in their gut microbiota compared with healthy individuals, including a notable reduction in microbiota diversity, a decrease in the abundance of Firmicutes, and significant increases in the abundances of Bacteroidetes and Actinobacteria (24). Studies of animal models have shown that the gut microbiota contributes to the onset of IBD, that germ-free mice receiving the transfer of IBD donor microbiota exhibit abnormal immune responses relative to those of germ-free mice receiving the transfer of healthy donor microbiota, and that the colonization of IBD microbiota can exacerbate colitis in mice (25). When determining the diagnosis of IBD, both UC and CD can be detected through an analysis of the patient’s fecal microbial composition. For example, some of the bacteria commonly found in the fecal microbiota of healthy individuals, including species such as *Eubacterium rectale*, *Bacteroides*, and *Faecalibacterium prausnitzii* (*F. prausnitzii*), typically comprising 40% of the total microbiota. However, in patients with CD or diarrhea, the abundance of these bacteria is often significantly reduced, if not completely absent (26).

**Table 1:** Changes in the gut microbiota observed in IBD patients.

Species affected	Changes in IBD	Model	Disease subtype	Reference
*Mucispirillum schaedleri*	Increased	Mice	CD	(143)
*Bifidobacterium breve, Clostridium symbiosum*	Increased	Human	UC	(144)
*Ruminococcus gnavus*	Increased	Human	Both UC and CD	(145)
*Actinomyces*, *Veillonella*, *Escherichia coli*	Increased	Human	CD	(146)
*Klebsiella*, *Streptococcus*	Increased	Human	UC	(40)
*Akkermansia*	Decreased	Mice	IBD	(147)
*Bacteroidetes*, *Firmicutes*	Decreased	Human	IBD	(148)
*Cyanobacteria*, *Flavobacterium*, *Oscillospira*	Decreased	Human	Both CD and UC	(149)
*Methanobrevibacter*, *Faecalibacterium*, *Anaerostipes*,	Decreased	Human	CD	(150)

Although whether gut microbiota dysbiosis in IBD patients is a cause or consequence of the disease remains unclear (27), gut microbiota dysbiosis plays a pivotal role in the pathogenesis and progression of IBD. For instance, in patients with IBD, the abundance of lactic acid-producing bacteria (such as *Lactobacillus*) often decreases, leading to a reduction in lactic acid levels and a change in intestinal pH, subsequently affecting the metabolism of intestinal substances (28). Patients with IBD exhibit a marked increase in the abundance of sulfate-reducing bacteria (e.g., *Desulfovibrio* spp.), which consequently increases hydrogen sulfide (H_2_S) production (29). H_2_S exerts cytotoxic effects on colonic cells, disrupts the intestinal mucosal barrier, and induces inflammation (30). Notably, gut microbiota dysbiosis can significantly impact the differentiation of immune cells, particularly Th17 cells. Th17 cells secrete proinflammatory cytokines such as interleukin-17 (IL-17), which can drive intestinal inflammation and the development of IBD (31). Moreover, gut microbiota dysbiosis leads to an imbalance in microbial metabolite levels. In IBD patients, the abundance of *F. prausnitzii*, a major butyrate-producing bacterium, is reduced, resulting in decreased levels of SCFAs (32). A meta-analysis revealed two Clostridia-derived biosynthesis-related gene clusters (BGCs). Subsequent research has shown that microbe-derived molecules can disrupt intestinal permeability and thereby exacerbate disease (33). A recent review comprehensively revealed that the effect of dysbiosis on inflammatory markers can not only aggravate the chronic inflammatory response but also participate widely in the pathological process of a variety of metabolic diseases (such as diabetes) (34).

The interplay between the gut microbiota and IBD is intricate and deeply interconnected, extending beyond straightforward cause-and-effect dynamics. An imbalance in the gut microbiota may influence the progression of IBD through various mechanisms. Therefore, correcting the imbalance of the gut microbiota becomes particularly critical in the holistic management of IBD, which suggests the feasibility of current gut microbiota-based diagnostic and therapeutic strategies for IBD.

## 5 Metabolites of the gut microbiota and IBD

Studies indicate that disruptions in the gut microbiota and abnormalities in the metabolic processes of these microbiota are associated with IBD development. The gut microbiota actively participates in host metabolic processes, generating diverse bioactive metabolites that contribute significantly to maintaining intestinal barrier integrity and immune homeostasis by supplying nutrients to intestinal epithelial cells (IECs) and directly or indirectly stimulating various receptors. For example, the gut microbiota is engaged in carbohydrate metabolism in the host *in vivo*, leading to the generation of SCFAs (35). Three categories of gut microbiota metabolites have been identified: compounds synthesized *de novo* by the microbiota; host-derived metabolites modified through microbial processing; and compounds generated from interactions between the microbiota and dietary components. Among these metabolites, SCFAs, BAs, and Trp metabolites are particularly relevant to IBD pathogenesis, and their disruption affects IBD disease progression in multiple ways.

### 5.1 The dysregulated metabolites: SCFAs, BAs, and Trp, in IBD

#### 5.1.1 Changes in SCFAs levels in patients with IBD

SCFAs are fatty acids with carbon chains containing 1 to 6 atoms, primarily acetic acid, propionic acid, and butyrate, produced via the anaerobic fermentation of indigestible carbohydrates by intestinal microbes. The Bacteroidetes phylum is mainly responsible for the production of most of the acetic and propionic acids, while the Firmicutes phylum mainly produces butyrate. A potential link has been observed between a marked reduction in the abundance of butyrate-producing *Roseburia hominis* bacteria in IBD patients and a lower level of SCFAs in their feces (36). Another study revealed greater instability in the fecal microbiota of individuals with IBD than in that of healthy subjects, along with a reduced abundance of the butyrate-producing species *F. prausnitzii* in their gut microbiota (32). Xu et al. (37) conducted a meta-analysis of 11 studies and revealed that compared with healthy individuals, patients with UC presented significantly reduced levels of total SCFAs, including acetate, propionate, and valerate, with these concentrations varying according to the disease status. Acetate and propionate levels were reduced in patients with active UC but unchanged in those in remission; butyrate levels were decreased in patients with active UC but increased in those in remission (37). Based on the quantitative analysis of SCFAs from different populations, the ratio of acetic acid to (propionic + butyric + isovaleric + valeric acid) yielded 92% sensitivity and 81% specificity in distinguishing healthy individuals from IBD patients based on fecal SCFA quantification (38). Based on these consistent changes, measuring SCFA levels is promising as a potential future tool for assessing disease stages and informing more precise treatment strategies, although the results require further validation in prospective clinical cohorts.

#### 5.1.2 Changes in BA levels in patients with IBD

BAs, the final products of cholesterol metabolism, facilitate lipid absorption in the digestive tract. BAs produced by the liver, termed primary bile acids, consist of cholic acid (CA) and chenodeoxycholic acid (CDCA), which are further transformed by the gut microbiota through dissociation and dehydroxylation to produce secondary bile acids, including deoxycholic acid (DCA), ursodeoxycholic acid (UDCA), and lithocholic acid (LCA). Therefore, alterations in the gut microbiota of IBD patients could be a significant factor contributing to their disrupted BA metabolism. A study of 103 patients with IBD showed that fecal samples from CD patients with dysbiosis exhibited notably lower concentrations of secondary bile acids, including DCA and LCA, whereas the levels of CA and its glycine and taurine conjugates tended to be enriched compared with those in CD patients without dysbiosis (39). Yang et al. (40) reported that the levels of primary bile acids, such as CA and taurocholic acid, were markedly higher in the feces of UC patients than in those of healthy controls, and their concentrations were positively correlated with the prevalence of bacteria such as *Enterococcus*, *Streptococcus*, and *Klebsiella*; in contrast, the concentrations of secondary bile acids, such as DCA and LCA, were significantly lower in UC patients than in healthy controls, and their levels were positively correlated with the abundance of probiotics such as *Butyricicoccus* and Clostridium cluster IV. Another study revealed that gut ecological dysregulation in IBD patients resulted in a reduced ratio between *F. prausnitzii* and *Escherichia coli*, allowing for reduced deconjugation of BAs, which resulted in a higher proportion of bound bile acids in the fecal matter (41). Analysis of fecal and serum BAs in children with IBD has demonstrated a strong correlation with disease activity, and the primary-to-secondary BA ratio in serum serves as a novel, excellent composite marker for stratifying IBD activity (42). Conversely, the gut microbiota composition is similarly affected by BAs. *In vitro* studies have shown that secondary bile acids (LCA and DCA) exhibit direct antifungal activity against *Candida albicans*, inhibiting the growth of *Candida albicans* and the formation of germ tubes, hyphae, and biofilms (43). Notably, experiments in mouse models demonstrated that dietary DCA supplementation led to marked increases in the abundances of intestinal *Parabacteroides* and *Bacteroides* while reducing the abundances of Lachnospiraceae and Ruminococcaceae (44).

#### 5.1.3 Changes in Trp levels in patients with IBD

Trp is a vital amino acid that is essential for the body. A reconstruction of metabolic models via multiomics approaches revealed that gut microbiota dysbiosis disrupts the cellular uptake and enzymatic processing of Trp (45). The gut microbiota is likely to influence the progression of IBD through Trp metabolism. After three years of continuous follow-up of 535 IBD patients, Trp metabolism increased in patients with active IBD, resulting in a notable reduction in serum Trp levels compared with those in controls; moreover, serum Trp levels were negatively correlated with disease activity, and Trp deficiency could be linked to further progression of IBD (46). In another study of patients with active and remission CD, the investigators measured serum levels of Trp and kynurenine (KYN), as well as the KYN/Trp ratio, in both patients with CD and healthy individuals and similarly observed significantly decreased serum Trp concentrations and an increased KYN/Trp ratio in individuals with clinically active CD compared with healthy controls and remission-phase patients. Notably, this ratio exhibited strong positive correlations with CD activity scores, the erythrocyte sedimentation rate (ESR), and C-reactive protein (CRP) levels (47). A study measuring the plasma KYN/Trp ratio in patients with active IBD demonstrated that this ratio may serve as a potential marker for assessing IBD disease severity (48). Trp metabolism is largely influenced by the gut microbiota, as the gut microbiota can convert Trp into indole, tryptamine (TA), and other indole metabolites through the action of tryptophanase and tryptophan decarboxylase. Studies have shown that the metabolites produced by Trp catabolism by the gut microbiota can positively affect the gut microbiota itself and the host and that the stability of the gut microbiota and its interaction with Trp metabolism play a vital role in maintaining intestinal function stability and preventing disease.

### 5.2 Key roles of SCFAs, BAs, and Trp metabolites in the gut

#### 5.2.1 Convergent mechanisms of SCFAs, BAs, and Trp metabolites in gut homeostasis

Beyond their quantitative changes, SCFAs, BAs, and Trp metabolites constitute a critical communication network between the host and gut microbiota, converging on three fundamental pillars of gut homeostasis. First, they collectively reinforce the intestinal epithelial barrier, a frontline defense mechanism achieved through the upregulation of tight junction proteins (TJPs) and an increase in transepithelial electrical resistance (TEER) (49–52). Second, they orchestrate innate immune responses by modulating the functions of classical innate immune cells such as macrophages, thereby fine-tuning intestinal inflammation (53–55). Finally, they critically shape adaptive immunity by regulating the delicate balance between proinflammatory and anti-inflammatory T-cell populations (54, 56, 57). Collectively, by fortifying the epithelial barrier, calibrating innate immunity, and directing adaptive responses, these three classes of metabolites function as indispensable pillars in the maintenance of gut homeostasis. The distinctive biological roles of each metabolite class are then defined by the specific receptors they engage and the unique downstream pathways they activate, which will be elaborated upon in the following sections.

#### 5.2.2 SCFAs and IBD

The consistently observed changes in SCFAs in the gut of IBD patients (as summarized in Section 5.1.1) have prompted extensive research into the functional consequences of an SCFA deficit, particularly regarding its impact on the intestinal barrier and intestinal immunity.

##### 5.2.2.1 Role of SCFAs in intestinal barrier maintenance

The intestinal barrier serves as the primary interface separating the host’s internal milieu from the luminal environment. SCFAs, particularly butyrate, play a distinct and crucial role in strengthening this barrier through multiple targeted mechanisms.

**Figure 1:**
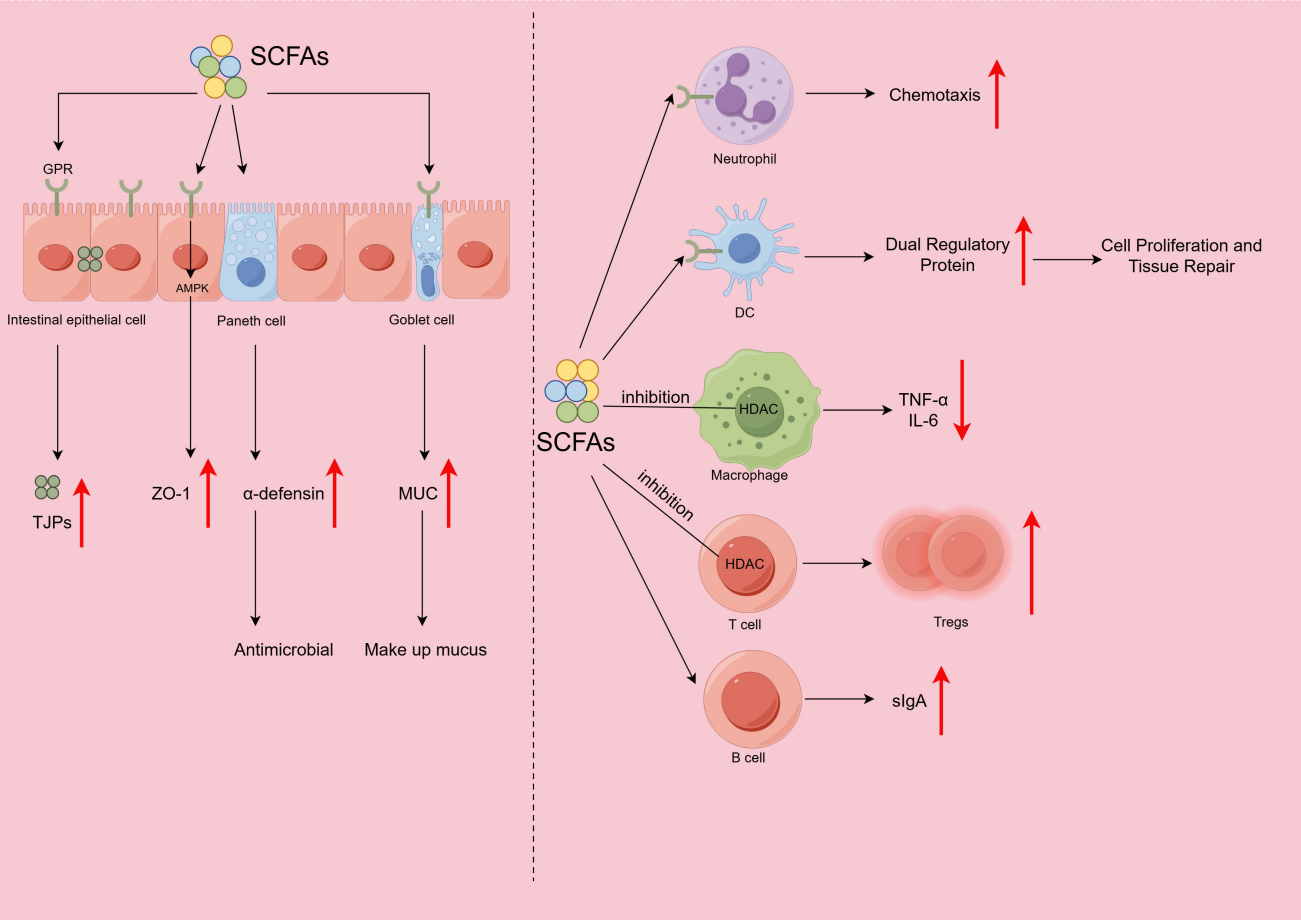
Mechanisms and roles of short-chain fatty acids (SCFAs) in intestinal barrier maintenance and immune regulation. The activation of G protein-coupled receptors (GPRs) on the surface of intestinal epithelial cells increases the expression of tight junction proteins (TJPs), such as claudin, occludin, and zonula occludens-1 (ZO-1). Activation of the AMP-activated protein kinase (AMPK) pathway increases ZO-1 expression. Paneth cells are stimulated to secrete α-defensins, and goblet cells produce mucin (MUC). GPR43 activation induces neutrophil chemotaxis, resulting in anti-inflammatory effects. GPR43 signaling regulates dendritic cell (DC) function and induces the expression of amphiregulin. Histone deacetylase (HDAC) activity is inhibited to reduce the production of proinflammatory cytokines (tumor necrosis factor-α [TNF–α] and interleukin-6 [IL-6]) by macrophages and promote Treg differentiation. Finally, the secretion of secretory immunoglobulin A (sIgA) by B cells is increased.

A key mechanism is an increase in tight junction integrity. Various TJPs, such as zonula occludens 1 (ZO-1), claudin, and occludin, are expressed in the intestinal epithelium. Claudin and occludin engage with the extracellular matrix, and ZO-1 acts as a connector for these proteins and regulates the composition of epithelial cell TJs, forming a physical barrier for IECs (58–60). Butyrate increases the synthesis of TJPs in the colon and maintains intestinal homeostasis (49) (**Figure 1**; **Table 2**). In animal models, isobutyrate has been shown to directly activate G protein-coupled receptor 109A(GPR109A), upregulate the expression of Claudin-1, and thereby improve intestinal barrier function (61). SCFAs promote the expression of ZO-1 in IECs by activating the AMP-activated protein kinase (AMPK) pathway, thereby increasing the TEER of epithelial cells and preserving the structural stability of the intestinal mechanical barrier (50).

**Table 2:** Comprehensive overview of key microbial metabolites.

Metabolites	Receptor(s)/mechanism	Principal cellular target	Exemplary effects	Evidence tier	Reference(s)
SCFAs	GPR activation	IECs	Secrete TJPs and increase TEER	Animal models	(49, 50)
	GPR41 activation	Paneth cells	Secrete α-defensin	Animal models and *in vitro* studies	(65)
	GPR43 activation	DCs	Express amphiregulin	Animal models	(69)
	GPR43 activation	Neutrophils	Improve phagocytic activity	Animal models	(70)
	HDAC inhibition	Macrophages	Improve antimicrobial activity	*In vitro* studies	(53)
	HDAC inhibition	T cells	Induce the production of both Teffs and Tregs	Animal models and *in vitro* studies	(74)
BAs	TGR5 activation	IECs	Secrete TJPs and increase TEER	*In vitro* studies	(51)
	FXR activation	IECs	Secrete TJPs and increase TEER	*In vitro* studies	(80)
	TGR5–cAMP–PKA pathway	Macrophages	Reduce NLRP3 and IL-6 expression	Animal models and *in vitro* studies	(81)
	FXR activation	Macrophages	Reduce NF-κB transcription and IL-6, IL-1β, and TNF-α levels	*In vitro* studies	(83)
	TGR5 activation	Macrophages	Increases the numbers of M2 macrophages and Tregs	*In vitro* studies	(54)
Trp	KYN–AHR pathway	IECs	Increases proliferation	*In vitro* studies	(88)
	KYN–AHR pathway	DCs	Increases IL-10 levels	*In vitro* studies	(89)
	KYN–AHR pathway	Macrophages	Reduces IL-6 levels	*In vitro* studies	(88)
	KYN–AHR pathway	HMuSCs	Increases TSG-6 levels	Animal models and *in vitro* studies	(90)
	KYN–AHR pathway	T cells	Induces the production of Tregs	*In vitro* studies	(57)
	Indole–AHR pathway	IECs	Secrete TJPs and increase TEER	*In vitro* studies	(52)
	Indole–AHR pathway	CD4^+^ IELs	Induces the production of CD4^+^/CD8^+^ IELs	Animal models	(94)
	Indole–AHR pathway	T cells	Induces the production of Tregs	Animal models	(96)
	Indole–PXR pathway	IECs	Reduces TNF-α levels	*In vitro* studies	(97, 98)
	5-HT–5-HTR2B pathway	DCs	Reduces IL-6 levels	*In vitro* studies	(101)
	5-HT-5-HTR	Macrophages	Increases the number of M2 macrophages	*In vitro* studies	(55)
	5-HT–5-HTR1A/5-HTR1B/5-HTR2A pathway	T cells	Increases proliferation and differentiation	*In vitro* studies	(103)
	5-HT–5-HTR1A pathway	B cells	Increases proliferation	*In vitro* studies	(102)

Beyond acting as physical barriers, SCFAs actively strengthen chemical defensive lines. The intestinal epithelium is composed of not only enterocytes but also specialized cell types, including goblet cells and Paneth cells (62). Goblet cells secrete a variety of intestinal mucins (MUCs) (58, 63). The results of animal studies, such as the work by Zhao et al. (64), suggest that SCFAs promote MUC secretion via GPR pathways, thereby enhancing intestinal resistance to colitis. The results from both animal models and *in vitro* experiments have verified that GPR41 expressed by Paneth cells can recognize butyrate and enhance chemical barrier function through the secretion of α-defensins, thus maintaining intestinal homeostasis (65).

In terms of regulating the microbial barrier, SCFAs lower the pH of the gut by dissociating and releasing H^+^, causing bacteria to expend a substantial quantity of ATP to actively expel H^+^ ions from their interior, which affects the energy needed for adequate growth and metabolic processes, resulting in energy competition (66). On the other hand, SCFAs also activate and regulate the host immune system, prompting host cells to produce antimicrobial peptides (67), thus preventing the biosynthesis of harmful bacteria, inhibiting their growth, and achieving a balanced intestinal microecology.

##### 5.2.2.2 Immunomodulatory mechanisms of SCFAs

SCFAs also modulate intestinal immunity, exerting potent anti-inflammatory effects by regulating the functions of both intrinsic and adaptive immune cells via mechanisms involving the activation of GPRs and the suppression of histone deacetylase (HDAC) activity.

The GPR43-dependent pathway is critical for innate immunity. A previous study indicated that supplementing the drinking water of a mouse model of DSS-induced colitis with acetate resulted in decreased intestinal inflammation, an increased colon length, and a decreased disease activity index, whereas mice lacking the GPR43 gene experienced no significant therapeutic benefit, suggesting that acetic acid may influence colitis via GPR43 (68). Studies using a mouse model revealed that butyrate regulates dendritic cells (DCs) through the GPR43/Blimp-1 pathway, inducing the expression of amphiregulin, which helps to stabilize the internal environment of the gut (69). SCFAs can also induce neutrophil chemotaxis and regulate phagocytic activity through activation of the GPR43 pathway, resulting in potential anti-inflammatory effects (70). Studies have shown that butyrate can effectively suppress macrophage activation by lipopolysaccharide (LPS) and reduce the production of large amounts of inflammatory mediators by macrophages (71). Butyrate suppresses the NLRP3 inflammasome signaling pathway, reduces the secretion of proinflammatory mediators such as caspase-1 and IL-1β, and thereby effectively inhibits the proinflammatory polarization of macrophages (72). Butyrate simultaneously reduces the generation of proinflammatory mediators, including IL-6, and prevents intestinal macrophages from overresponding to microbial stimuli (73). *In vitro*, butyrate induces the differentiation of monocyte-derived macrophages and increases their intrinsic antimicrobial activity through HDAC inhibition, according to a recent study (53).

Furusawa et al. (56) used both *in vitro* cultures and mouse models treated with butyrate and observed increased histone H3 acetylation at the Foxp3 locus, the induced the differentiation of regulatory T cells (Tregs) in the colon, and subsequent anti-inflammatory effects that ameliorated intestinal inflammation in IBD models during adaptive immunity. Through the potent suppression of the HDAC pathway and modulation of the mTOR–S6K pathway, SCFAs can induce the production of effector T cells (Teffs) and Tregs (74). Upon antigen stimulation, B cells proliferate and differentiate into a multitude of plasma cells, which synthesize and secrete large quantities of antibodies (e.g., Ig) to participate in immune protection through the blood circulation. Research has shown that SCFAs increase the levels of sIgA-coated bacteria, promote sIgA secretion, and increase blood IgA levels (75).

Studies of the effects of traditional Chinese medicine on mouse colitis models have demonstrated that Sishen Pill and Tongxieyaofang (SSP-TXYF) exert therapeutic effects on IBD by modulating the gut microbiota, thereby increasing the levels of propionic and butyrate. These changes lead to the acetylation of hypoxia-inducible factor-1α (HIF-1α), ultimately regulating inflammation. This evidence suggests that SSP-TXYF may function through the gut microbiota–propionic and butyrate–HIF-1α axis, indicating a novel approach for the treatment of IBD (76). Certain studies have also indicated that SCFAs can have a proinflammatory effect that may be related to variations across disease models, the dose and manner in which SCFAs are administered, and the exacerbation of inflammation due to the local accumulation of large numbers of neutrophils. Therefore, while extensive *in vitro* and animal model data compellingly illustrate the mechanisms by which SCFAs may ameliorate IBD, their efficacy and optimal application in human patients require further validation through large-scale clinical trials.

#### 5.2.3 BAs and IBD

The observed alterations in the BA composition and their correlations with specific microbial taxa in IBD patients (as detailed in Section 5.1.2) have prompted investigations into the functional consequences of dysregulated BA metabolism. A key question is how these quantitative changes translate into qualitative shifts in host signaling.

##### 5.2.3.1 Role of BAs in intestinal barrier maintenance

BAs influence IBD progression primarily by engaging specific receptors, notably Takeda G protein-coupled receptor 5 (TGR5) and the farnesoid X receptor (FXR), to preserve intestinal barrier integrity.

**Figure 2:**
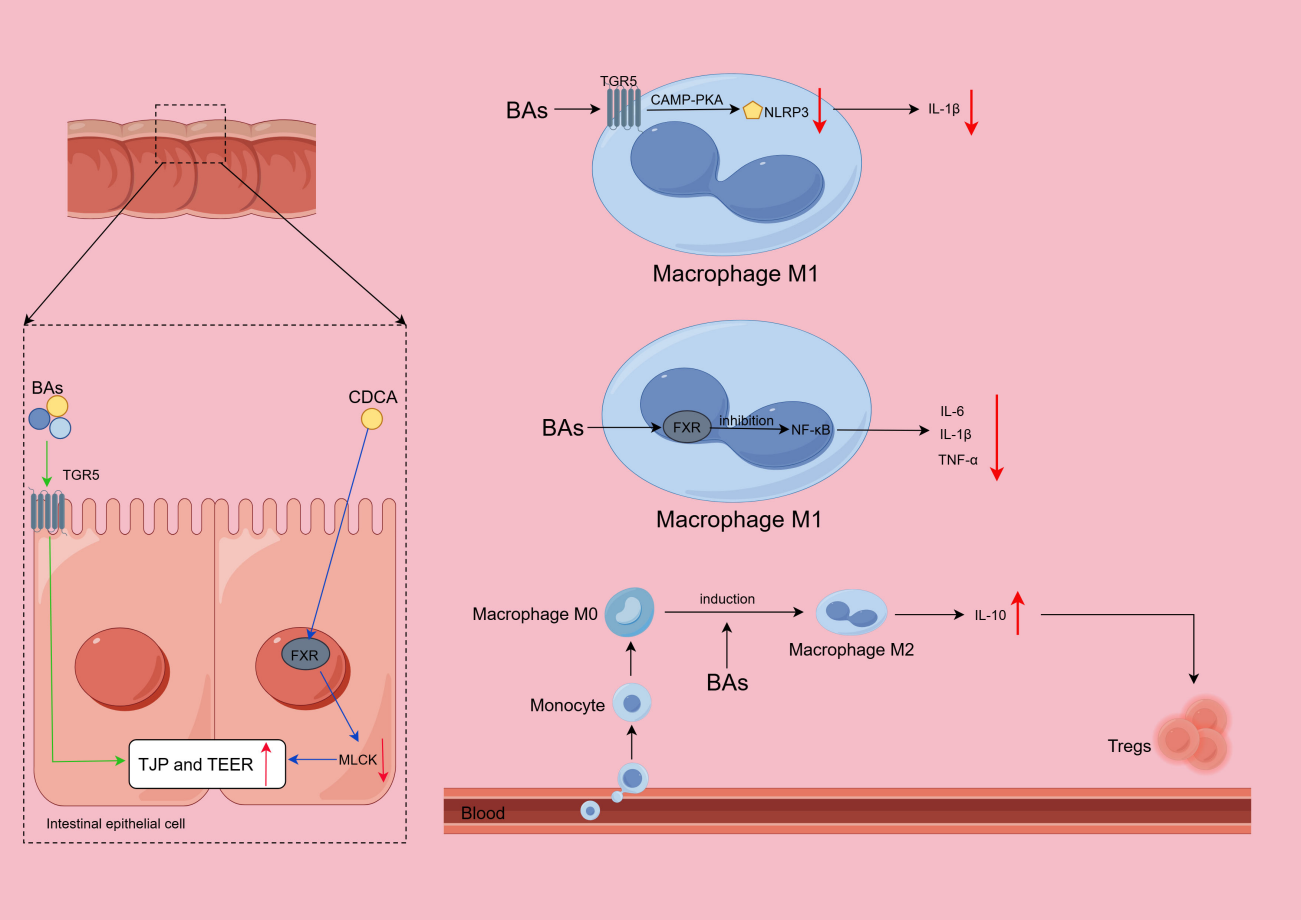
Mechanisms and roles of bile acids (BAs) in intestinal barrier maintenance and immune regulation. BAs activate Takeda G protein-coupled receptor 5 (TGR5) to attenuate the decrease in transepithelial electrical resistance (TEER) and upregulate tight junction proteins (TJPs). Chenodeoxycholic acid (CDCA) binds to the farnesoid X receptor (FXR), inhibiting the myosin light-chain kinase (MLCK) pathway, thereby preventing a reduction in TEER and increasing TJP expression. Upon binding to TGR5, BAs trigger the TGR5-cyclic adenosine monophosphate (cAMP)–protein kinase A (PKA) signaling pathway to suppress NLRP3 inflammasome formation, thereby reducing interleukin-1β (IL-1β) secretion. FXR activation inhibits the production of proinflammatory factors (such as IL-6, IL-1β, and tumor necrosis factor-α [TNF-α]) mediated by the nuclear factor kappa-B (NF-κB) pathway. BAs induce an anti-inflammatory phenotype in macrophages, thereby modulating immune responses.

TGR5 is a crucial receptor for BAs. Sorrentino et al. (77) showed that both BAs and TGR5 agonists stimulate the development of intestinal organoids and that mice lacking TGR5 display a more severe form of colitis than those with intact TGR5. The deletion of TGR5 leads to severe colonic histopathological changes in mice, in which the molecular structure of intercolonic TJs is disrupted, thereby increasing intestinal permeability (78). An *in vitro* study using Caco-2 cells has shown that TGR5 activation inhibits the LPS-induced decrease in TEER and promotes the production of TJPs, such as claudin-1, thereby improving barrier function (51) (**Figure 2**). Research in IEC lines revealed that normal concentrations of DCA increase the levels of cyclooxygenase-2 (COX-2) and the secretion of prostaglandin (PG) through the epidermal growth factor receptor–extracellular signal-regulated kinase (EGFR–ERK) pathway, which subsequently promotes the proliferation and division of IECs while preserving the structural integrity of the intestinal epithelial barrier (79).

FXR is another important high-affinity receptor for BAs. Mechanistic cell-based studies have shown that the FXR-dependent pathway mediates the inhibition of the LPS-induced activation of the myosin light-chain kinase (MLCK) pathway by CDCA. In these experiments, CDCA supplementation was shown to reverse the LPS-induced decreases in TEER and TJP expression, thereby attenuating intestinal barrier damage (80).

##### 5.2.3.2 Immunomodulatory mechanisms of BAs

In addition to maintaining and restoring the integrity of the intestinal epithelial barrier, BAs also exert anti-inflammatory effects through both TGR5 and FXR.

An *in vitro* study using macrophages revealed that the activation of TGR5 by BAs induces a 'mixed phenotype' dominated by the anti-inflammatory M2 phenotype, which produces IL-10 and may promote Treg cell activation (54). By performing an *in vivo* experiment in mice, Guo et al. (81) observed that BAs bind to TGR5 as ligands and inhibit the formation of the NLRP3 inflammasome through the TGR5–cyclic adenosine monophosphate (cAMP)–protein kinase A (PKA) signaling pathway, thereby decreasing the release of proinflammatory factors, including IL-1β. The activation of TGR5 also inhibits the activation of nuclear factor kappa-B (NF-κB) and inhibits its transcriptional function, thereby exerting an anti-inflammatory effect (82). These studies suggest that TGR5 is a potential target for effectively attenuating the intestinal inflammatory response in IBD.

Vavassori et al. (83) reported that the deletion of the FXR gene exacerbated colitis in mice, whereas treatment with 6-ethylchenodeoxycholic acid (6E-CDCA) alleviated colitis severity and reduced the activation of immune cells and the expression of proinflammatory cytokines in wild-type (WT) mice, but similar effects were not detected in FXR-deficient (FXR-/-) mice. The activation of FXR in macrophages suppresses the NF-κB pathway, reducing the generation of inflammatory factors such as IL-6, IL-1β, and tumor necrosis factor-α (TNF-α) (83). In mice with colitis, treatment with obeticholic acid (INT-747), a targeted FXR receptor agonist, significantly inhibited intestinal epithelial permeability, reduced intestinal epithelial goblet cell loss, and attenuated inflammation. An analysis of cytokines revealed that the levels of cytokines that promote inflammation, such as TNF-α, decreased (84). Collectively, these findings from preclinical models establish FXR as a crucial regulator of intestinal innate immunity and homeostasis. Moreover, BAs can stimulate additional nuclear receptors, including the pregnane X receptor (PXR), vitamin D receptor (VDR), and constitutive androstane receptor (CAR).

Overall, IBD patients often have abnormal BA metabolism, and preclinical studies have elucidated promising pathways for treating IBD through BAs and their receptors. However, translating these findings into clinical practice necessitates a deeper understanding of BA biology in humans with IBD and the development of safe, effective receptor-targeted agents.

#### 5.2.4 Trp metabolism and IBD

**Figure 3:**
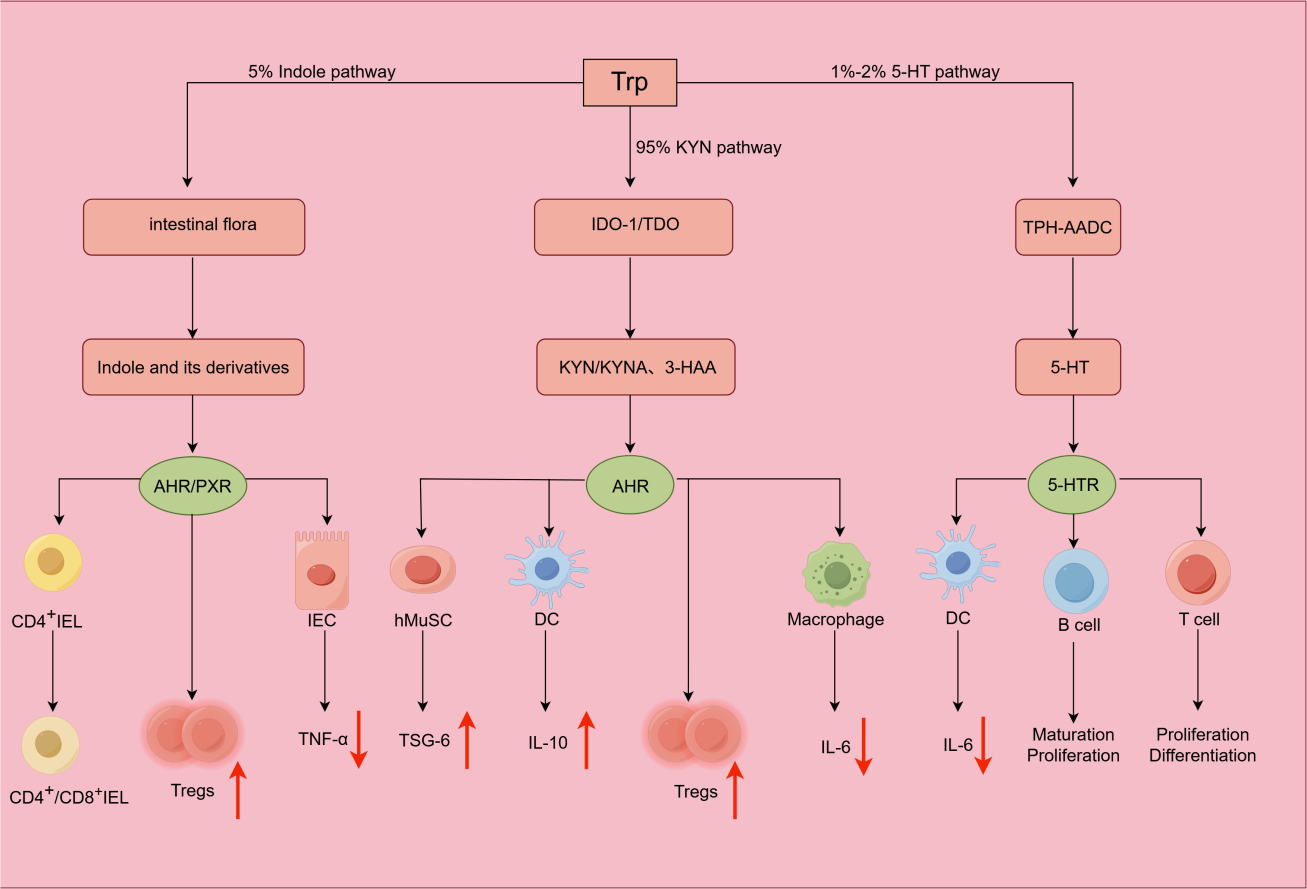
Mechanisms and roles of tryptophan (Trp) metabolites in intestinal immune regulation. Most Trp (~95%) is metabolized via the kynurenine (KYN) pathway, where indoleamine 2,3-dioxygenase 1 (IDO-1) or tryptophan 2,3-dioxygenase (TDO) catalyze the conversion of Trp to KYN and its downstream metabolites; these metabolites can activate the aryl hydrocarbon receptor (AHR), which regulates intestinal immunity. A minor fraction of Trp (~5%) is metabolized by the gut microbiota into indole and its derivatives; these microbial metabolites can engage AHR or pregnane X receptor (PXR), balancing immune responses. Less than 2% of Trp is sequentially converted to 5-hydroxytryptophan (5-HTP) by tryptophan hydroxylase (TPH) and then decarboxylated to 5-hydroxytryptamine (5-HT) via aromatic L-amino acid decarboxylase (AADC); 5-HT exerts immunomodulatory effects by binding to 5-hydroxytryptamine receptors (5-HTRs) on immune cells.

The observed systemic alterations in the levels of Trp and its metabolites in IBD patients (Section 5.1.3) are driven by distinct biochemical pathways within the gut. These pathways and their specific bioactive products must be delineated to understand the functional effects of these changes. In the gut, Trp is involved in three metabolic pathways, namely, the KYN axis, indole pathway, and 5-hydroxytryptamine (5-HT) pathway. Among these pathways, the KYN axis is quantitatively dominant, accounting for approximately 90–95% of systemic Trp metabolism, while the indole and 5-HT pathways constitute minor fractions (**Figure 3**).

##### 5.2.4.1 The KYN axis

Among the various pathways, the KYN axis is the most critical for Trp metabolism, and more than 90% of Trp in the human body is converted to KYN and downstream products such as 3-hydroxyanthranilic acid (3-HAA) and kynurenic acid (KYNA) by tryptophan 2,3-dioxygenase (TDO) and indoleamine-2,3-dioxygenase (IDO). TDO and IDO function as key regulatory enzymes in this metabolic process, controlling the rate at which the pathway progresses. TDO is activated by glucocorticoids, whereas IDO is primarily modulated by IFN-γ (85). Sofia reported that IDO1 expression is increased in the colonic mucosa during active UC and that KYNA levels are increased relative to those of Trp, which correlate with endoscopic severity in UC patients (86).

The most distinctive role of the KYN axis is its function as a primary source of endogenous ligands for the aryl hydrocarbon receptor (AHR). AHR, a key ligand-activated molecule, is ubiquitously expressed in IECs and immune cells. In patients with IBD, AHR activity can significantly mediate inflammatory responses and alleviate intestinal injury (87). *In vitro* studies using human colonic epithelial cells have shown that KYN promotes wound healing and helps preserve intestinal barrier integrity (88). A body of *in vitro* evidence was used to further elucidate the immunomodulatory mechanisms of KYN and its downstream products. KYN promotes IL-10 secretion by DCs (89). In macrophages, KYN inhibits the expression of IL-6 induced by LPS (88). Research has shown that KYN, or its metabolite KYNA, can increase the expression of tumor necrosis factor-stimulated gene 6 protein (TSG-6) in human muscle satellite cells (hMuSCs), thereby exerting anti-inflammatory effects and alleviating symptoms of IBD (90). Recent research has shown that KYN can inactivate the NF-κB signaling pathway and inhibit NLRP3 inflammasome formation, thereby reducing intestinal inflammation (91). In addition, 3-HAA has immunomodulatory effects, decreasing the synthesis of proinflammatory mediators by inhibiting phosphatidylinositol 3-kinase (PI3K) activity and NF-κB activity. KYN is also capable of inducing Treg generation in an AHR-dependent manner (57).

##### 5.2.4.2 The indole pathway

Approximately 5% of Trp in the human body is directly converted to indole and indole derivatives by the gut microbiota. The evidence suggests that intestinal microorganisms such as *Clostridium perfringens* can convert Trp into TA and indole-3-propionic acid (I3P). These metabolites are further processed into indole derivatives, including indole acetaldehyde (IAId), indole acetic acid (IAA), and indole ethanol (IEt) (92). *Streptococcus pepticus* can convert Trp to indole-3-pyruvic acid (IPA).

At physiological concentrations, indole derivatives are capable of promoting mucus secretion by IECs (93). These bioactive derivatives maintain intestinal homeostasis primarily through AHR and PXR. For example, in a Caco-2/HT29 cell coculture model, I3P robustly enhanced intestinal barrier integrity by increasing TEER and increasing the expression of TJPs (claudin-1, occludin, and ZO-1) while simultaneously strengthening the mucus layer via the upregulation of MUC2 and MUC4 expression. Moreover, I3P mitigated the LPS-induced inflammatory response by suppressing the expression of proinflammatory factors (52). The intestinal epithelial integrity and barrier function of IPA-treated mice were restored via the PXR-dependent downregulation of TNF-α expression and remodeling of apical junctional complexes, collectively strengthening the intestinal barrier (94, 95).

Concurrently, these indole derivatives orchestrate local and systemic immune responses. Studies using mouse models have shown that indole-3-lactic acid (ILA), which is produced by *Lactobacillus reuteri*, can induce CD4^+^ intraepithelial lymphocytes (IELs) to become CD4^+^/CD8^+^ double-positive IELs, a population associated with the suppression of intestinal inflammation (94). Shen et al. (96) employed whole-genome sequencing to profile the gut microbiota of experimental mice and found that IAA increased Treg proliferation through the activation of the AHR pathway. IAA also suppressed the production of proinflammatory cytokines (IL-6, IL-17A, IL-23, and TNF-α) and stimulated the release of the anti-inflammatory cytokine IL-10. *In vitro* experiments have indicated that IPA can reduce TNF-α production in IECs by binding to and activating PXR (97, 98).

##### 5.2.4.3 The 5-HT pathway

Studies conducted in the past century have shown that patients with IBD have notably lower levels of 5-HT in the gut than healthy individuals (99). In the gut, intestinal chromaffin cells are responsible for the synthesis of more than 90% of 5-HT. Tryptophan hydroxylase (TPH) mediates the hydroxylation of Trp to form 5-hydroxytryptophan (5-HTP), which undergoes decarboxylation via aromatic L-amino acid decarboxylase (AADC) to produce 5-HT. The gut microbiota directly influences 5-HT levels *in vivo* by regulating key zymogens. Furthermore, certain metabolites generated by the gut microbiota have been discovered to influence 5-HT production in studies in which SCFAs trigger free fatty acid receptors in enterochromaffin cells and upregulate the expression of TPH1, in turn increasing 5-HT production. BAs can also activate TGR5 in enterochromaffin cells and promote 5-HT secretion (100).

Beyond its role as a neurotransmitter, 5-HT functions as a critical immunomodulator. In cultures of DCs, 5-HT, which acts on the 5-HTR2B receptor, can inhibit IL-6 expression (101). Similarly, research in macrophage models has indicated that 5-HT modulates immune responses by suppressing the LPS-induced expression of proinflammatory mediators while upregulating the expression of genes linked to M2 polarization (55). In the context of adaptive immunity, evidence from *in vitro* lymphocyte cultures has shown that 5-HT promotes B-cell proliferation via the 5-HTR1A receptor (102) and that signaling through receptors, including 5-HTR1A, 5-HTR1B, and 5-HTR2A, can facilitate the clonal expansion and differentiation of T cells (103).

Metabolites from the three Trp metabolic pathways exert both direct and indirect regulatory effects on intestinal homeostasis and inflammatory processes, critically influencing the pathogenesis and clinical trajectory of IBD. Nevertheless, current studies are mainly based on experimental models such as mice, which have limitations and a wide variety of Trp derivatives. Further clinical studies are still needed to elucidate the mechanistic links between Trp metabolites and human physiological states, as well as their underlying molecular mechanisms.

## 6 The gut microbiota and its metabolite in IBD treatment

Because a complete cure for IBD is unavailable, the treatment of CD and UC aims to alleviate symptoms and improve quality of life. Current therapeutic agents include aminosalicylates, thiopurines, glucocorticoids, and immunosuppressive agents aimed at controlling gut inflammation and preventing complications. However, a variety of factors are closely associated with the development of IBD, including genetics, environmental factors, immunological factors, and the gut microbiota, and single treatments have limited effectiveness. Dysbiosis of the gut microbiota and imbalances in microbial metabolites may serve as critical drivers of IBD pathogenesis. Consequently, treatment based on the gut microbiota and metabolites has emerged as a pivotal research focus, representing one of the most promising innovative approaches for IBD management.

### 6.1 Microecological agents

**Figure 4:**
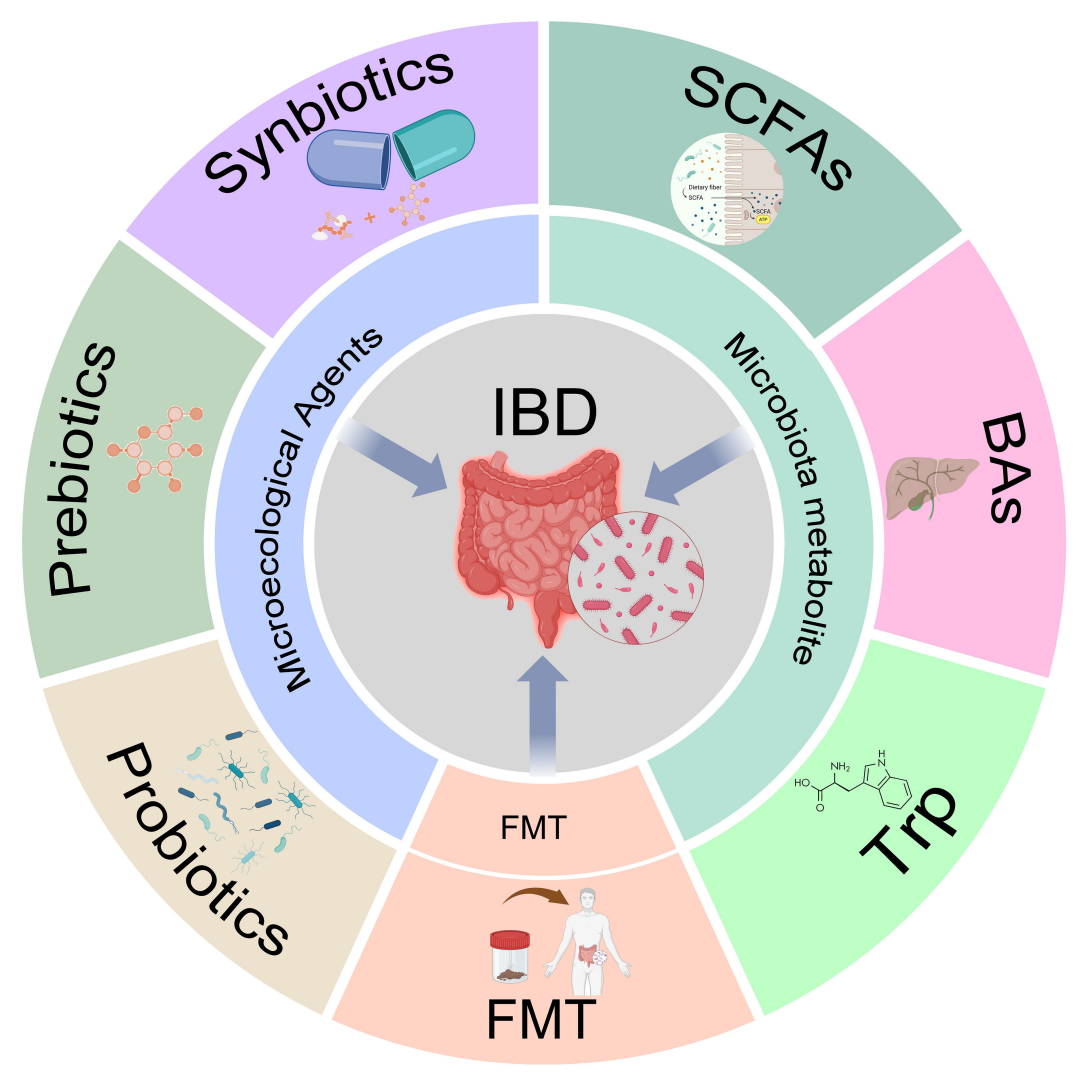
The gut microbiota and its metabolite in inflammatory bowel disease (IBD) treatment. This figure summarizes the methods of treating IBD through probiotics, prebiotics, synbiotics, fecal microbiota transplantation (FMT), short-chain fatty acids (SCFAs), bile acids (BAs), and tryptophan (Trp).

Microecological agents (also known as microecological regulators) improve intestinal resistance to pathogenic bacteria and correct symptoms such as diarrhea by adjusting and re-establishing the balance of the gut microbiota. Clinically, they mainly include probiotics, prebiotics, and synbiotics (**Figure 4**).

#### 6.1.1 Probiotics

Probiotics include live beneficial microorganisms that confer health benefits by colonizing certain areas and dynamically modulating local microbial ecosystems. They can be either single strains or combinations of multiple strains, with *Bifidobacterium*, *Lactobacillus*, and *Bacillus* being some of the most common probiotics. The administration of an appropriate amount of probiotics can promote a healthier balance of the gut microbiota and increase the integrity of the intestinal epithelial barrier to fortify its defense against luminal threats, regulate intestinal immunity, and alleviate disease. Probiotics enhance mucosal barrier function by inhibiting epithelial cell apoptosis and upregulating TJP expression, which synergistically restore gut barrier integrity and promote tissue repair (104). *In vitro* studies have demonstrated that probiotics exert immunomodulatory effects by activating natural killer (NK) cells and amplifying the effector functions of other immune cells, coupled with the selective induction of anti-inflammatory cytokine synthesis, thereby mitigating intestinal inflammatory responses (105). In murine models, both viable *Lactobacillus plantarum* AN1 and its heat-inactivated counterpart showed therapeutic efficacy by ameliorating colon shortening, preserving the mucosal architecture, attenuating splenomegaly, and enriching natural lactic acid bacteria. Notably, this microbial enrichment had a synergistic effect with the administration of *Lactobacillus plantarum* AN1 cells on IBD (106). *Lactobacillus plantarum* BD7807 mitigates high-fat diet-induced metabolic dysregulation and intestinal dysfunction in mice by activating the SCFAs-GPR43 pathway (107). An evaluation of a 6-week intervention with a probiotic mixture containing 9 *Lactobacillus* and 5 *Bifidobacterium* strains in UC patients revealed significant improvements in the partial Mayo score, stool frequency, and overall assessment (108). In the future, we hope to further explore the components, optimal dosage, and optimal treatment duration of probiotic preparations through multicenter and large-sample verification.

#### 6.1.2 Prebiotics

Prebiotics are organic compounds that, while indigestible by the host, selectively increase the metabolic activity and growth of beneficial bacteria, thereby improving intestinal health and supporting overall well-being. Prebiotics commonly include substances such as inulin, oligo-lactose, lactulose, and oligo-galactose. By stimulating the production of probiotic bacteria such as *Lactobacillus* and *Bifidobacterium*, prebiotics promote the production of lactic acid and SCFAs, lowering the pH of the colon, thus reducing the proliferation of harmful anaerobic bacteria, and protecting the intestinal tract (109). In one study, prebiotics substantially ameliorated gut inflammation in patients with IBD. For example, Koleva et al. (110) showed that dietary supplementation with inulin and oligofructose attenuated colonic inflammatory responses in rats. Notably, rats administered inulin exhibited significantly reduced intestinal levels of the proinflammatory mediator IL-1β, providing mechanistic evidence for its anti-inflammatory action within the gastrointestinal niche (110). Park et al. (111) demonstrated that the administration of oligogalactose products to a mouse model of experimental DSS-induced colitis resulted in fewer inflammatory symptoms, such as weight loss and colon shrinkage, and reduced levels of proinflammatory cytokines such as IL-6 and TNF-α. Research using mouse models has shown that inulin consumption can affect the composition of the gut microbiota and the production of its metabolites. These metabolic alterations are closely tied to type 2 inflammation, which is characterized by increased IL-33 production, ILC2 activation, and an increase in the number of eosinophils (112). Recent research using animal models has revealed for the first time an anticolitis mechanism involving fructo-oligosaccharide (FOS), which promotes the production of IAA and IPA by improving the imbalance of the gut microbiota and regulating microbial Trp metabolism, thus activating the AHR/IL-22 axis (113). Prebiotic supplementation over 8 weeks significantly reduced the clinical activity index and improved the remission rate compared with the placebo in a double-blind randomized controlled trial involving 40 patients with mild-to-moderate UC (114). A systematic review of 17 studies showed that prebiotics have therapeutic potential as safe and effective dietary interventions to induce and maintain the remission of UC (115). At present, most studies on the efficacy of prebiotics on IBD are still in the animal experimental stage, and more clinical trials are needed to verify these findings.

#### 6.1.3 Synbiotics

Synbiotics have evolved beyond mere combinations of probiotics and prebiotics. As defined by the International Scientific Association for Probiotics and Prebiotics, they are now described as ‘a mixture of active microorganisms and substrates selectively utilized by host microorganisms to provide health benefits to the host’ (116). Studies have shown that synbiotics are more effective than either probiotics or prebiotics alone at improving gut inflammation. In experimental colitis models, groups receiving *Bifidobacterium infantis*, xylooligosaccharide (XOS) supplementation, or their synbiotic combination all presented reduced disease activity index (DAI) and pathological scores; notably, the synbiotic regimen demonstrated superior efficacy, uniquely increasing the expression of the anti-inflammatory cytokine IL-10 (117). Research using mouse models has indicated that *Bifidobacterium infantis* and 3'-sialyllactose (3'-SL) synergistically treat DSS-induced colitis. Compared with single-component treatments, the synbiotic approach more effectively alleviated colitis symptoms through increased restoration of gut microbiota homeostasis and SCFA levels. Moreover, synbiotic treatment significantly mitigates inflammatory responses by increasing the production of the anti-inflammatory cytokines IL-10 and TGF-β (118). After administering synbiotics to colitis mice, the levels of pro-inflammatory cytokines secreted by Th17 cells were reduced, while the levels of anti-inflammatory cytokines produced by Treg cells were elevated. Concurrently, a significant increase in SCFAs and BAs was observed (119). In a randomized trial, compared with a placebo, an 8-week synbiotic intervention significantly reduced systemic inflammation in patients with mild to moderate UC, as evidenced by reduced serum CRP levels and sedimentation rates (120).

Although a significant body of research has indicated that microecological agents contribute to alleviating IBD (121), their therapeutic efficacy is still limited, and the exact mechanisms of action remain unclear. At present, microecological agents are mainly used as adjuvant therapy, and further exploration of more effective agents is still needed.

### 6.2 FMT

Recently, FMT has become a focus of interest as a novel therapeutic approach. FMT helps restore the gut microbiota balance and rebuild the gut ecosystem by transferring the functional microbiota from donors in good health to the patient’s intestines through capsules or enemas. In recent years, numerous studies have been conducted to assess the effectiveness and safety of FMT in the treatment of IBD, and some advancements have been achieved. A meta-analysis of cohort studies by Chen et al. (122) revealed that FMT has superior therapeutic efficacy for IBD patients with *Clostridium difficile* infections (CDIs), achieving dual outcomes of gut microbiota restoration and the remission of symptoms. Another study also confirmed that FMT increases the number and diversity of beneficial bacteria in IBD patients while decreasing intestinal barrier damage and inhibiting the secretion of IL-8 and monocyte chemoattractant protein 1 (MCP-1) (123). In a randomized, double-blind, placebo-controlled study, patients with UC were divided into two groups: one group was administered oral lyophilized FMT, while the other received a placebo. Following an eight-week treatment period, 53% of patients (8 of 15) in the FMT group achieved remission, a rate that was substantially higher than the 15% remission rate (3 of 20) observed in the placebo group. Additionally, compared with the placebo group (85%), the FMT group experienced a lower rate of adverse events (67%) during the induction phase (124). Another study of patients suffering from active UC who underwent treatment using FMT capsules revealed a reduction in the diversity of fungi in the patients’ feces, along with an improvement in the fungal composition. In addition, significant reductions in the levels of pathogenic organisms, such as *Candida*, were also observed (125). A study evaluating FMT therapy in CD patients involved administering treatments to 25 participants at three-month intervals. After three months of initial treatment, 17 of 25 patients, or 68.0%, showed signs of improvement, and 13 of 25, or 52.0%, achieved clinical remission. The rates of sustained clinical remission were maintained at 48.0% (12/25) at six months and 32.0% (8/25) at one year and decreased slightly to 22.7% (5/22) at 18 months of continued FMT therapy. Importantly, no serious adverse events associated with FMT were documented during the study (126). Initial research suggests that FMT may offer certain therapeutic advantages for individuals with IBD, but the duration of its effect is short, and the long-term remission rate gradually decreases. Moreover, a multicenter randomized trial revealed that FMT failed to induce clinical and endoscopic remission in patients with mild-to-moderate CD (127). Therefore, many issues still need to be addressed, such as how to select the best donor for fecal bacteria transplantation, determine the optimal method and frequency of transplantation, improve the survival rate of transplanted microorganisms, and explore the factors affecting the efficacy of the treatment. These issues require extensive experimental research to improve the effectiveness and safety of FMT.

### 6.3 Microbiota metabolite-based IBD therapy

As previously noted, three classes of metabolites produced by the gut microbiota significantly contribute to regulating the microbial composition, preserving intestinal barrier integrity, and orchestrating immune homeostasis. These findings suggest that we can consider metabolites as potential therapeutic molecules. By regulating the levels of these metabolites and taking full advantage of their unique biological properties, the effective reduction of intestinal inflammation and improved intestinal health may be possible. This line of thinking provides a new direction for future therapeutic strategies and opens up broader prospects for studying the relationship between the gut microbiota and metabolites.

#### 6.3.1 SCFAs in IBD therapy

SCFAs, particularly butyrate, have long been the focus of research on IBD treatment. In one study, 49 IBD patients were administered exogenous butyrate preparations for two months, and the findings revealed that butyrate had a notable effect on the gut microbiota of patients with UC and CD, increasing the population of bacteria capable of producing butyrate (128). Di Sabatino et al. (129) treated 13 patients with mild-to-moderate CD for 8 weeks with 4 g/d butyrate tablets. During the course of treatment, one patient withdrew from the study, and of the remaining 12 patients, seven achieved complete remission, two achieved partial remission, and three showed no clinical improvement. However, collectively, the patients exhibited marked improvements in endoscopic and histological scores, as well as substantial reductions in white blood cell counts, the sedimentation rate of erythrocytes, and mucosal NF-κB and IL-1β levels, underscoring the effectiveness of SCFAs for mild-to-moderate CD (129). A double-blind randomized controlled study showed that butyrate is expected to be an effective adjuvant therapy for active UC based on its role in reducing the levels of inflammatory biomarkers, upregulating circadian clock genes, and improving sleep quality and quality of life (130). A medicine made from *Abelmoschus manihot* (AM) is widely used in China. It can increase the diversity of the gut microbiota, particularly by increasing the number of microbes that produce SCFAs. This increase in SCFA production further stimulates Treg cell generation while suppressing the production of Th17 cells, effectively mitigating DSS-induced colitis in mice (131). To date, sufficient support and evidence concerning the use of SCFAs as treatments in clinical practice have not been obtained, and their efficacy and safety still require more extensive clinical validation. Future research and clinical practice will further reveal the potential role of SCFAs in IBD treatment and provide a more comprehensive scientific basis for their application.

#### 6.3.2 BAs in IBD therapy

Oral treatment with UDCA and tauro-ursodeoxycholic acid (TUDCA) effectively reduced inflammatory symptoms in mice with DSS-induced colitis, such as by mitigating body weight loss, while the Firmicutes-to-Bacteroidetes ratio, which is linked to colitis, was restored to normal levels (132). In addition, as mentioned earlier, INT-747, which activates the bile acid receptor FXR, significantly reduced inflammation in mice. With the increasing understanding of the BA receptor, the development of BA receptor agonists and antagonists has become an important direction in the current research on IBD therapy. One study investigated the role of nelumal A, a novel FXR agonist, in the prevention of colitis and colorectal carcinogenesis. The findings revealed that nelumal A increased the expression of TJPs, antioxidant enzymes, and FXR target genes, and inhibited the expression of genes involved in hepatic bile acid synthesis. Additionally, it reduced colitis symptoms and decreased the risk of colitis-associated cancers in mice, highlighting its therapeutic potential for IBD and chemopreventive effects on colorectal cancer (133). A recent study using mouse models demonstrated that the combination of TUDCA and emodin significantly alleviated colitis severity. Further *in vitro* analysis revealed that it maintained TJP levels, restored intestinal barrier integrity, and promoted the recovery of gut microbiota diversity, suggesting a novel therapeutic approach for colitis (134). Further mechanistic research is essential to fully elucidate the function of BAs in the intestine. The interplay between the gut microbiota and BAs presents novel opportunities for IBD therapy. Despite advancements in elucidating the interactions between the gut microbiota and BAs, their therapeutic use requires further rigorous investigation to bridge the existing knowledge gaps.

#### 6.3.3 Trp and its derivatives in IBD therapy

Although Trp and its derivatives have received less attention in IBD research, they still have great potential applications. Recent research has demonstrated that IAA not only significantly reduces the production of proinflammatory mediators (such as IL-6 and IL-8) in animal models, resulting in clear anti-inflammatory effects, but also effectively reduces the anxiety-like behavior of animals. These findings highlight the potential of IAA as a novel therapeutic agent for IBD that is capable of addressing both intestinal inflammation and the associated psychological burden (135). In a murine colitis model, tryptophan supplementation (100 mg/kg) significantly reduced serum TNF-α levels and histological inflammatory scores while increasing IL-10 levels (136). As previously noted, serum Trp levels were reduced in patients with active CD compared with healthy subjects and those in remission, while the KYN/Trp ratio was increased, and the ratio was positively related to disease activity, the rate of erythrocyte sedimentation, and CRP levels in CD patients. Thus, Trp and its derivatives can be used as biomarkers for diagnosing and evaluating disease severity. In addition, the ability of Trp to regulate the gut microbiota and its potential for immunomodulation and controlling inflammation represent promising new directions for IBD therapy and the development of novel treatments. In the future, Trp metabolic interventions based on the regulation of the gut microbiota may emerge as a novel therapeutic strategy for IBD.

Collectively, microecological agents, FMT, and microbiota metabolite-based therapies hold substantial translational potential for IBD management, but their clinical application necessitates addressing critical safety and quality considerations: strain specificity (efficacy and safety vary markedly across microbial strains or metabolite formulations), determination of the optimal dosage (e.g., 10^7^–10¹^0^ CFUs for probiotics, standardized transplant frequency for FMT), and stringent manufacturing or processing consistency (e.g., probiotic viability preservation, FMT donor screening standardization, and control of metabolite purity). Clinical trials of these strategies typically employ stratified patient selection (by disease subtype [UC *vs*. CD], activity [mild/moderate/severe], and prior treatment history) and adopt well-established IBD endpoints, including clinical remission (assessed via Mayo score for UC or CDAI for CD), endoscopic healing (Mayo endoscopic subscore ≤1), and histological improvement (e.g., reduced mucosal inflammation and restored epithelial barrier structure) to ensure a rigorous evaluation and realistic expectations for outcomes. At present, the application of metabolomics and multiomics technology has led to the identification of metabolic characteristics related to disease subtypes and treatment outcomes (137), providing a concrete framework for evaluating these novel strategies to move the field from aspirational promise to actionable evidence.

## 7 Future perspectives and challenges

Looking ahead, the central challenge lies in advancing from correlative observations to establishing causal mechanisms and translating these findings into reliable therapies, considering the significant impact of interindividual metabolic variation on treatment efficacy. Emerging technologies, particularly multiomics integration and single-cell microbiome analysis, will be critical in deciphering functional interactions between specific microbial consortia and the host (138). Genetically engineered microorganisms represent a promising strategy for precision immunotherapy (139, 140), while nanotechnology may overcome limitations associated with the delivery of metabolite-based interventions (141, 142). Future therapeutic frameworks should prioritize personalized approaches, shifting from single-strain probiotics toward rationally designed microbial consortia and metabolite-centric regimens. Ultimately, overcoming hurdles in standardization, safety, and patient stratification will be essential to fully realizing the potential of microbiota-targeted therapies for IBD.

## 8 Summary and outlook

This review highlights the critical role of gut microbiota dysbiosis and the resulting imbalance of key microbial metabolites—SCFAs, BAs, and Trp metabolites—in the pathogenesis of IBD. These metabolites are essential for maintaining intestinal barrier integrity and immune homeostasis, and their disruption actively drives disease progression. We explore how this understanding is translated into novel therapeutic strategies, such as microecological agents, FMT, and metabolite-based interventions. While promising, these approaches require further validation and personalization to overcome current challenges in efficacy and standardization, representing the next frontier in IBD treatment.
